# Advancing towards HIV-1 remission: Insights and innovations in stem cell therapies

**DOI:** 10.46439/stemcell.5.020

**Published:** 2024

**Authors:** Aditi Chatterjee, Aerielle Matsangos, Olga S. Latinovic, Alonso Heredia, Giovannino Silvestri

**Affiliations:** 1 Department of Medicine, School of Medicine, University of Maryland, MD, 21201, USA; 2 Marlene and Stewart Greenebaum Comprehensive Cancer Center, University of Maryland, Baltimore, MD, 21201, USA; 3 Department of Microbiology and Immunology, School of Medicine, University of Maryland, Baltimore, MD, 21201, USA; 4 Institute of Human Virology, University of Maryland, Baltimore, MD, 21201, USA

**Keywords:** HIV-1, cART, Stem cell therapy

## Abstract

Human immunodeficiency virus type 1 (HIV-1) continues to pose a significant global health challenge despite advances in combined antiretroviral therapy (cART), which has transformed HIV-1 infection from a fatal disease to a manageable chronic condition. However, cART is not curative, and its long-term use is associated with challenges such as pill burden, drug toxicities, and the emergence of drug-resistant viral strains. The persistence of active viral reservoirs necessitates lifelong treatment, highlighting the need for alternative therapeutic strategies capable of achieving HIV-1 remission or cure. Stem cell therapy has emerged as a promising approach to address these challenges by targeting latent viral reservoirs, restoring host immune function, and potentially achieving sustained viral suppression in the absence of cART. This review critically evaluates current scientific literature on stem cell therapies for HIV-1, focusing on three major approaches: *1) hematopoietic stem cell transplantation (HSCT)*, *2) gene therapy, and 3) cell-based immunotherapies*. Each approach is examined in terms of its underlying mechanisms, clinical feasibility, recent advancements, and associated challenges. Furthermore, future research directions are discussed, emphasizing the optimization of the current treatment protocols, enhancement of safety and efficacy, and the importance of large-scale clinical trials with different cohorts (different HIV clades, different genders of participants, and pediatric HIV) to evaluate long-term outcomes that include effective and scalable HIV cure challenges. Collaborative efforts across multidisciplinary fields are needed to overcome existing barriers so to realize the full therapeutic potential of stem cell-based approaches for developing an effective and scalable remission or cure strategies.

## Introduction

HIV-1 remains a significant global health challenge, with approximately 38 million people living with HIV (PLWH) worldwide and 3,600 new infections daily (2022), of which about 50% occur in Sub Saharan Africa [1]. Despite substantial progress in treatment and prevention efforts, HIV-1 continues to exert a profound impact on public health systems globally. The implementation of cART has revolutionized the management of HIV-1 infection, transforming what was once a fatal disease into a chronic, manageable condition [2]. cART effectively suppresses viral replication by targeting various stages of the HIV life cycle, thereby preventing disease progression, transmission and restoring immune function in treated PLWH [3].

However, despite the success of cART in improving patient outcomes, several challenges persist. A primary concern is the lifelong nature of cART, requiring strict adherence to daily medication regimens. The pill burden associated with cART can lead to challenges in patient adherence, impacting treatment efficacy and long-term health outcomes [4]. Moreover, the psychological and logistical burdens of continuous medication can affect patients’ quality of life and contribute to treatment fatigue over time [5]. The most recent success with the long acting injectables is a big step forward and it has been reported in 2024 demonstrating their superiority to the standard of care (SOC).

In addition to adherence challenges, long-term cART use is associated with potential drug toxicities and side effects. Antiretroviral drugs can exert serious adverse effects on multiple organ systems, ranging from mild gastrointestinal discomfort to severe complications such as cardiovascular disease, renal dysfunction, and metabolic abnormalities [6,7]. These toxicities not only affect patient well-being, but also necessitate regular monitoring and management, adding complexity to HIV SOC.

Other critical issues in HIV-1 management are the development of drug resistance and viral rebound upon cessation of cART HIV-1’s Env high genetic diversity across all subtypes and rapid replication rate facilitate the emergence of drug-resistant viral strains, significantly compromising the effectiveness of cART regimens [8]. The need for alternative treatment strategies that can address drug-resistant HIV variants and overcome persistent viral reservoirs are therefore paramount in achieving sustained viral suppression and improving long-term outcomes for PLWH [9].

Moreover, despite effective viral suppression with cART, HIV-1 establishes latent reservoirs within long-lived CD4^+^ T cells and other immune cell subsets including various tissues [10]. These active viral reservoirs harbor integrated proviral DNA that remains transcriptionally silent and is not susceptible to current antiretroviral drugs [11]. Latently infected cells can persist for years, posing a significant barrier to achieving a cure for HIV1. As previously mentioned, the reactivation of latent reservoirs upon treatment interruption leads to viral rebound, necessitating lifelong cART to maintain viral suppression [12]. cART is often combined with other treatments such as broadly neutralizing antibodies (bnAbs) that target assessable parts of the HIV Env successfully while killing the HIV-1 infected cells. bnAbs have dual roles of neutralization and cytotoxicity, making them superior to cART for preventions and treatment strategies. In response to these challenges, there is growing interest in exploring novel therapeutic approaches that have the potential to achieve HIV-1 remission or even a functional cure. Among these innovative strategies, stem cell therapy has emerged as a promising avenue for HIV-1 treatment. Stem cell therapies encompass a range of techniques aimed at harnessing the regenerative potential of stem cells to restore immune function and eliminate or control HIV-1 reservoirs [13].

**Figure 1.**
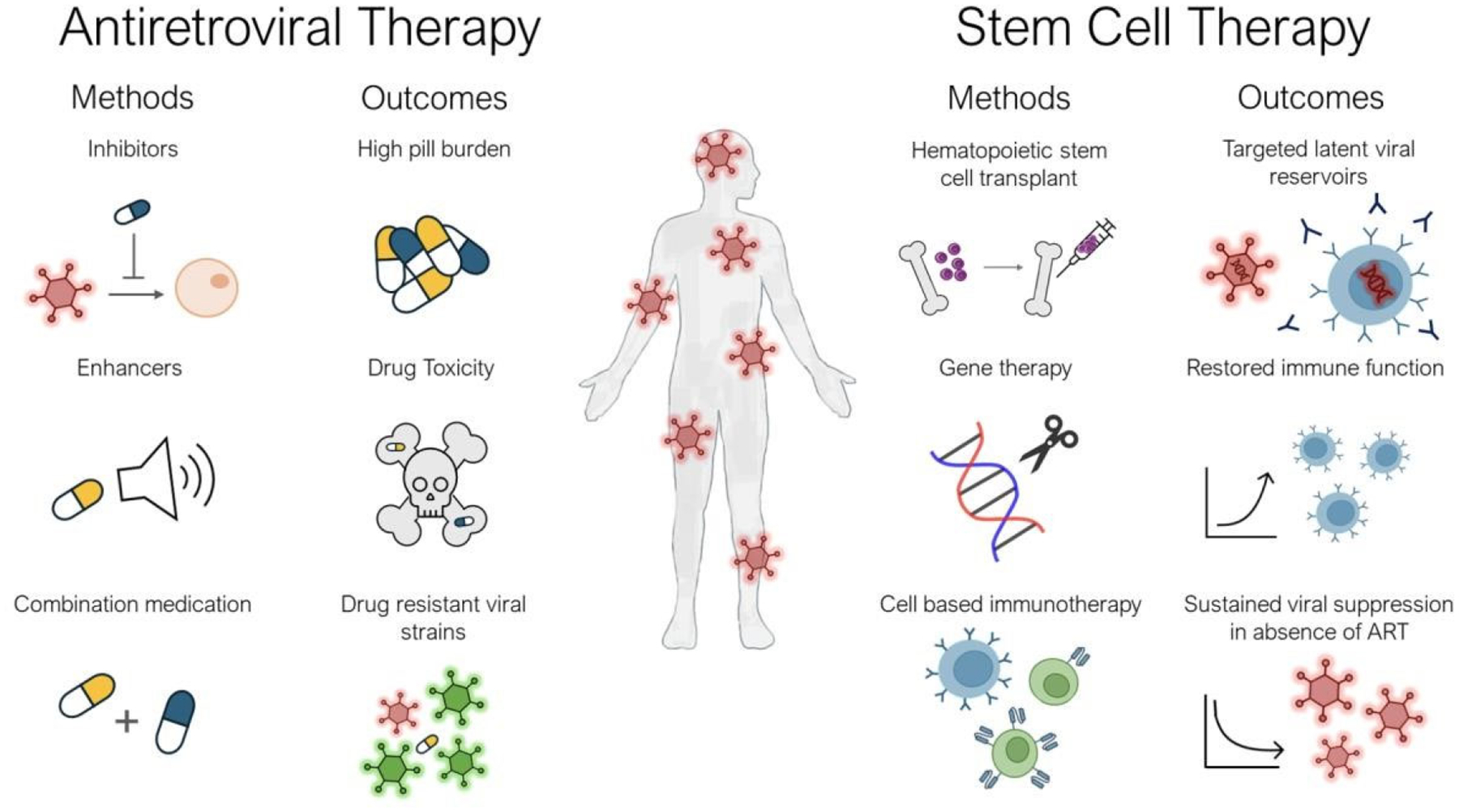
Differences between methods and outcomes for HIV-infected individuals treated with ART or Stem cell therapy. HIV-infected individual (center), ART methods and outcomes (left), and stem cell therapy methods and outcomes (right). HIV virus depicted as red pronged hexagon and generic human cell depicted as nude toned sphere. Antiretroviral medication depicted as blue and yellow pills. Drug resistant HIV strain represented as green pronged hexagon. Purple cells indicate hematopoietic stem cells. Generic immune cell with antibodies, T-cells with multiple CARs and a single CAR illustrated as blue cell and green cells, respectively. Targeted latent viral reservoirs depicted as generic blue immune cells with red HIV DNA.

Stem cell therapy offers several distinct advantages in the context of HIV-1 (Figure 1). It has the potential to replace the depleted CD4^+^ T cells and other immune cell populations that are severely affected by HIV-1 infection [14]. By replenishing the immune system with healthy, functional immune cells, stem cell transplantation could enhance immune competence and improve the body’s ability to combat HIV-1 [15]. Furthermore, advancements in gene therapy have enabled the modification of stem cells to render them resistant to HIV-1 infection or enhance their capacity to target and eliminate HIV-infected cells [16] (Figure 1).

Among the various forms of stem cell therapy, hematopoietic stem cell transplantation (HSCT) has garnered significant attention. HSCT involves the infusion of multipotent hematopoietic stem cells, typically derived from bone marrow, peripheral blood, or umbilical cord blood, to reconstitute the recipient’s immune system [17]. The success of HSCT in achieving long-term HIV-1 remission was notably demonstrated in the cases of the “Berlin Patient” and the “London Patient.” Timothy Ray Brown, the Berlin Patient, underwent HSCT with donor cells possessing a naturally occurring mutation in the CCR5 gene, which encodes a co-receptor used by HIV-1 for cell entry [18] (Figure 1). Following HSCT, Brown achieved sustained viral remission without the need for cART, providing compelling evidence of the potential of HSCT to cure HIV-1 [18].

In addition to HSCT, gene therapy approaches hold promise for enhancing the effectiveness of stem cell therapies in HIV-1 treatment. Gene editing technologies, such as CRISPR-Cas9, offer the possibility of precisely modifying the genetic material of stem cells to disrupt HIV-1 proviral DNA or introduce protective genetic modifications that confer resistance to HIV-1 infection [19]. These advances in gene therapy could revolutionize the field by creating a renewable source of HIV-resistant immune cells that could potentially eradicate or control viral reservoirs more effectively than current therapies [20].

Cell-based immunotherapies, including chimeric antigen receptor (CAR) T-cell therapy, represent another innovative approach in HIV-1 treatment. CAR T-cell therapy involves genetically engineering patients’ T cells to express receptors that recognize and target HIV-1-infected cells, thereby enhancing the immune response against the virus [21]. Preliminary studies have shown promising results in preclinical and early clinical trials, suggesting that CAR T-cell therapy could complement existing treatments by specifically targeting residual HIV-infected cells that evade traditional therapies [22] (Figure 1). This review critically evaluates the current scientific literature on the use of stem cell therapies, including HSCT, gene therapy, and cell-based immunotherapies, for the treatment of HIV-1. By examining the latest advancements, challenges, and future directions in these emerging therapeutic strategies, we aim to provide a comprehensive overview of their potential to achieve HIV-1 remission or cure.

Understanding the strengths and limitations of these innovative approaches is essential for guiding future research efforts and optimizing clinical outcomes in the pursuit of a definitive cure for HIV-1.

## Hematopoietic Stem Cell Transplantation

Hematopoietic stem cell transplantation (HSCT) stands at the forefront of innovative approaches for treating HIV-1, offering a potential pathway towards sustained remission or even a functional cure. This procedure involves the infusion of hematopoietic stem cells (HSCs), which possess the capacity to differentiate into various blood cell types, thereby restoring hematopoiesis in patients with hematological disorders such as leukemia, lymphoma, and certain immunodeficiencies. In the context of HIV-1, HSCT presents a unique opportunity to replace the host immune system with donor-derived cells that are intrinsically resistant to HIV-1 infection, primarily due to genetic mutations such as the CCR5 delta-32 deletion [23].

The CCR5 delta-32 mutation results in a truncated CCR5 protein that is not expressed on the surface of immune cells, rendering them resistant to HIV-1 entry via the CCR5 coreceptor. The groundbreaking cases of Timothy Ray Brown, known as the “Berlin Patient,” and the “London Patient” exemplify the potential of HSCT in achieving long-term HIV-1 remission. Timothy Ray Brown, diagnosed with both HIV-1 and acute myeloid leukemia (AML), underwent HSCT in 2007 with HSCs from a CCR5 delta-32 homozygous donor. Following transplantation, he ceased antiretroviral therapy and has remained free of detectable HIV-1 for over a decade, demonstrating the feasibility of achieving HIV-1 remission through HSCT from a CCR5-deficient donor [17].

Similarly, the “London Patient,” who received HSCT from a CCR5 delta-32 homozygous donor in 2019, has maintained undetectable HIV-1 viral load after discontinuing ART, further supporting the reproducibility of this approach [18]. Further investigations through case reports and small cohort studies have reinforced the pivotal role of the CCR5 delta32 mutation in achieving sustained HIV-1 suppression post-HSCT [13]. Studies involving patients transplanted with HSCs from donors lacking the CCR5 mutation have shown transient reductions in HIV-1 reservoirs but no sustained remission, underscoring the importance of donor selection for optimal outcomes in HIV-1 treatment strategies [23] (Table 1).

The mechanisms underlying HIV-1 remission following HSCT are multifaceted. Central to this approach is the complete replacement of the host immune system with donor-derived cells, which are not only resistant to HIV-1 infection but also possess the potential to actively clear HIV-1-infected cells through immune-mediated mechanisms [23]. Graftversus-host disease (GVHD), a complication of HSCT where donor immune cells attack host tissues, has been implicated in the eradication of residual HIV-1-infected cells. The conditioning regimens administered prior to HSCT, which typically include myeloablative chemotherapy and/or radiation, serve dual purposes of eliminating malignant cells (in cases of concurrent malignancies) and significantly reducing the HIV-1 reservoir by targeting infected cells, thereby contributing to the reduction in viral load [14].

Despite the promising outcomes observed in select cases, HSCT for HIV-1 is not without significant challenges and risks. Transplant-related morbidity and mortality remain substantial concerns, with complications such as infections, organ toxicity, and GVHD posing serious risks to patient health [16]. GVHD, while potentially beneficial in terms of HIV-1 eradication, necessitates long-term management with immunosuppressive drugs to mitigate its adverse effects, thereby increasing susceptibility to infections and other complications [24]. Lifelong immunosuppressive therapy is often required post-HSCT to prevent graft rejection, adding complexity to patient care and underscoring the need for personalized treatment approaches tailored to individual patient needs [25] (Table 1). A critical limitation of current HSCT approaches for HIV-1 is the availability of suitable donors with the CCR5 delta-32 mutation [26]. This mutation occurs predominantly in individuals of Northern European descent, presenting challenges in finding compatible donors, particularly for ethnically diverse populations. Addressing this limitation requires expanding donor registries globally and exploring alternative strategies to engineer HIV1-resistant cells through advanced gene-editing technologies [27].

Recent advancements in gene editing, particularly CRISPR-Cas9 technology, hold promise for overcoming the limitations associated with donor availability in HSCT for HIV1 [28]. Researchers are actively investigating ways to modify autologous HSCs *ex vivo* to confer resistance to HIV-1 infection by introducing the CCR5 delta-32 mutation or other protective genetic alterations. This approach aims to create a personalized treatment option where a patient’s own modified stem cells can be reinfused to establish an HIV-1resistant immune system, potentially obviating the need for donor-derived cells and reducing the risk of transplant-related complications [29].

In addition to gene editing strategies, ongoing research is exploring alternative conditioning regimens aimed at minimizing the toxicity associated with traditional myeloablative approaches [26]. Non-myeloablative or reduced-intensity conditioning regimens are being investigated to achieve sufficient engraftment of donor cells while reducing the overall burden of transplant-related morbidity and mortality [16]. Combination therapies involving latency-reversing agents and immune checkpoint inhibitors are also under scrutiny to enhance the clearance of latent HIV-1 reservoirs and achieve sustained remission post-HSCT [24] (Table 1).

Looking forward, future directions in HSCT for HIV-1 treatment necessitate expanding donor pools through international collaborations and optimizing gene-editing techniques to create HIV-1-resistant cells from a broader range of donors [25]. Personalizing conditioning regimens and post-transplant care based on individual patient characteristics and disease factors will be crucial in improving treatment outcomes and minimizing complications associated with HSCT [27]. Large-scale clinical trials are imperative to systematically evaluate the efficacy, safety, and long-term durability of HSCT for HIV-1 across diverse patient populations, providing robust evidence to guide clinical practice and facilitate broader implementation of this potentially transformative therapeutic approach [28].

Furthermore, combining HSCT with innovative therapeutic strategies could potentially enhance its effectiveness. For example, incorporating latency-reversing agents that can activate and expose latent HIV-1 reservoirs to immune clearance, in conjunction with HSCT, may offer a synergistic effect in eradicating the virus [29]. Additionally, the integration of immune checkpoint inhibitors, which can enhance the immune response against HIV-1-infected cells, could further improve the outcomes of HSCT by facilitating the clearance of residual infected cells that might otherwise persist [30] (Table 1).

The exploration of novel biomarkers to monitor the success of HSCT in real-time is also a critical area of research [14]. Biomarkers that can accurately reflect the status of HIV-1 reservoirs and immune reconstitution post-transplantation would enable clinicians to tailor treatment plans more effectively and predict potential complications earlier. Advanced imaging techniques and molecular assays are being developed to provide more precise and timely assessments of the viral and immunological landscape in patients undergoing HSCT [31].

Finally, patient education and support are essential components of successful HSCT for HIV-1 [16]. Given the complexity and risks associated with this treatment, comprehensive patient counseling and continuous psychological support are vital to help patients navigate the treatment journey. Informed patients who are actively engaged in their care are more likely to adhere to post-transplant regimens and report any adverse effects promptly, thereby improving overall outcomes.

## Gene Therapy

Gene therapy approaches for HIV-1 have garnered considerable attention as potential strategies to disrupt viral replication or enhance host immunity through the genetic modification of target cells, such as CD4^+^ T cells or hematopoietic stem cells (HSCs) [32–35]. One of the most prominent gene therapy strategies involves engineering cells to express a mutated form of the CCR5 co-receptor (CCR5 delta-32) that confers resistance to HIV-1 entry, as evidenced by the success of the Berlin Patient [13]. This mutation renders the CCR5 protein non-functional, thereby preventing HIV-1 from utilizing this receptor to infect cells. The Berlin Patient’s case provided a significant proof of concept, demonstrating that genetic modification could result in durable remission of HIV1. Recent advancements in gene-editing technologies, particularly CRISPR-Cas9, have facilitated precise modifications to the CCR5 gene in autologous CD4^+^ T cells or HSCs [34,35]. Clinical trials utilizing CCR5-modified autologous CD4^+^ T cells have shown promising results, with several studies reporting reduced viral reservoirs and prolonged viral suppression in treated patients [32,35–38]. For example, a phase I clinical trial involving the infusion of CCR5-modified CD4^+^ T cells into HIV-1 patients demonstrated not only the feasibility of the approach but also the potential for achieving sustained viral suppression without continuous antiretroviral therapy [32]. Similarly, another trial involving the use of zinc finger nucleases (ZFNs) to disrupt the CCR5 gene in autologous CD4^+^ T cells showed a significant reduction in viral load and an increase in CD4^+^ T cell counts, highlighting the therapeutic potential of this strategy [36]. However, the efficiency and specificity of gene editing techniques remain critical challenges in gene therapy for HIV-1. Off-target effects, where unintended genetic modifications occur, can lead to deleterious consequences, including the potential for oncogenic transformations or other adverse effects [34]. Researchers are actively working to improve the precision of gene-editing tools, employing strategies such as high-fidelity variants of CRISPR-Cas9 and optimized delivery methods to enhance targeting accuracy and minimize off-target modifications [34,35,37]. For instance, the development of base editors and prime editors represents a significant advancement, offering the ability to make single-base changes or insertions with greater specificity compared to traditional CRISPR-Cas9 systems [38]. Another major challenge is the immune response against genetically modified cells. The immune system may recognize the modified cells as foreign, leading to their elimination and reducing the overall efficacy of the therapy [34]. Strategies to mitigate this include transiently suppressing the immune system during the initial phase of gene therapy to allow the modified cells to engraft and proliferate [34]. Additionally, researchers are exploring the use of “stealth” modifications that evade immune detection, as well as engineering cells to express immunomodulatory molecules that can dampen immune responses [34]. Despite the success in modifying CCR5, the persistence of viral reservoirs remains a significant barrier to achieving a complete cure for HIV-1. Viral reservoirs, primarily composed of latently infected cells, can evade immune detection and standard antiretroviral therapies, leading to viral rebound once treatment is discontinued [34]. To address this, combination approaches are being investigated, where gene therapy is paired with latency-reversing agents (LRAs) that aim to “shock and kill” latent HIV-1 [34]. This approach involves using LRAs to reactivate latent virus, making infected cells visible to the immune system or other therapeutic agents, which can then target and eliminate these cells [34]. Clinical trials combining CCR5-modified cells with LRAs are ongoing, with the goal of achieving more robust reductions in viral reservoirs [34]. In addition to targeting CCR5, researchers are exploring other genetic targets and strategies to enhance host immunity against HIV-1. For example, gene editing approaches aimed at enhancing the expression of antiviral proteins or modifying immune cells to better recognize and attack HIV-1-infected cells are under investigation [32]. One such strategy involves engineering T cells to express chimeric antigen receptors (CARs) that specifically target HIV-1-infected cells [32]. CAR-T cell therapy, which has shown remarkable success in treating certain cancers, is being adapted for HIV-1 treatment. Preliminary studies have demonstrated that CAR-T cells can effectively target and kill HIV-1-infected cells, reducing viral load and potentially contributing to viral eradication [32]. The scalability and cost-effectiveness of gene therapy approaches remain significant barriers to widespread implementation. The complex and resource-intensive nature of gene-editing procedures, including the need for personalized manufacturing of genetically modified cells, limits the accessibility of these therapies to a broader patient population [34]. Efforts are underway to streamline the production process, improve the efficiency of gene editing, and reduce costs through automation and standardization [34,35,37]. Advances in delivery systems, such as viral vectors and non-viral methods, are also being explored to enhance the scalability and affordability of gene therapies [34]. Furthermore, regulatory and ethical considerations play a crucial role in the development and deployment of gene therapy for HIV-1. Ensuring the safety and efficacy of these therapies through rigorous clinical testing and adhering to ethical guidelines is paramount [34]. Public acceptance and trust in gene therapy will also be essential for its successful integration into mainstream medical practice. Engagement with patient communities, transparent communication about the benefits and risks of gene therapy, and addressing ethical concerns are vital components of this process [34]. In conclusion, gene therapy represents a promising frontier in the quest for an HIV-1 cure. The success of the Berlin Patient has spurred significant advancements in gene-editing technologies and has demonstrated the potential for achieving durable viral remission through genetic modification [13]. While challenges such as off-target effects, immune responses, and the persistence of viral reservoirs remain, ongoing research and technological innovations hold promise for overcoming these obstacles. Combination approaches, improved precision of gene-editing tools, and strategies to enhance scalability and costeffectiveness are key areas of focus. As the field continues to evolve, gene therapy has the potential to transform the landscape of HIV-1 treatment, offering hope for a functional cure and improved quality of life for individuals living with HIV-1 [32,34,35,37].

## Cell-Based Immunotherapies

Cell-based immunotherapies for HIV-1 aim to harness and enhance the body’s natural immune response to target and eliminate HIV-infected cells. These therapies typically involve the manipulation and infusion of immune cells such as cytotoxic T lymphocytes (CTLs), natural killer (NK) cells, and dendritic cells. Among the most studied approaches are the adoptive transfer of *ex vivo*-expanded HIV-specific CTLs [39] and the engineering of chimeric antigen receptor T cells that are designed to recognize and attack HIV infected cells [16].

HIV-specific CTLs are a type of immune cell that can recognize and kill virus-infected cells. In preclinical studies and early-phase clinical trials, the adoptive transfer of *ex vivo*-expanded HIV-specific CTLs has shown promise in controlling viral replication and delaying disease progression [39]. For instance, a study involving the infusion of autologous HIV-specific CTLs into patients on antiretroviral therapy demonstrated that these cells could persist *in vivo* and exhibit antiviral activity [39]. These findings suggest that CTLs can potentially be used to maintain viral suppression and contribute to immune control of HIV-1.

CAR T cell therapy, which has been revolutionary in the treatment of certain cancers, is also being explored for HIV-1. CAR T cells are genetically engineered to express receptors that can specifically target and bind to HIV antigens on the surface of infected cells [16]. This targeted approach enables CAR T cells to recognize and kill HIV-infected cells with high specificity. Early-phase clinical trials have shown that CAR T cells targeting HIV can reduce viral load and enhance immune control. For example, a clinical trial using CAR T cells engineered to target the HIV envelope glycoprotein gp120 demonstrated reduced HIV DNA levels in patients, indicating a potential reduction in viral reservoirs [16]. Despite these promising developments, several challenges remain. One significant hurdle is the identification and isolation of potent antiviral immune cells [40]. Not all patients possess naturally occurring HIV-specific CTLs with sufficient potency to control the virus. Moreover, the expansion of these cells *ex vivo* to clinically relevant numbers without losing their functional capacity is a technical challenge [40].

Researchers are exploring ways to optimize the selection and expansion processes, including the use of cytokines and other growth factors to enhance the proliferation and function of these cells [40].

The persistence and trafficking of infused cells *in vivo* is another critical challenge [41]. For cell-based immunotherapies to be effective, the infused cells must not only survive but also migrate to sites of HIV replication, including lymphoid tissues and other reservoirs [41]. Strategies to enhance the *in vivo* persistence of these cells include the genetic modification of T cells to express anti-apoptotic genes or the use of combination therapies that create a more favorable environment for their survival and function [41]. Additionally, advancements in imaging technologies are aiding in tracking the distribution and persistence of infused cells in patients, providing valuable insights for optimizing therapy [41].

Overcoming immune evasion mechanisms employed by HIV-1 is another area of intense research [42]. HIV-1 has evolved various strategies to evade immune detection, including downregulation of major histocompatibility complex (MHC) molecules and the establishment of latent reservoirs [42]. Innovative approaches to counteract these mechanisms include combining cell-based therapies with latency-reversing agents that reactivate latent HIV, making infected cells visible to the immune system [42]. Furthermore, researchers are investigating the use of immune checkpoint inhibitors, which can block inhibitory pathways that suppress the immune response, thereby enhancing the activity of HIV-specific T cells [42].

Concerns regarding potential off-target effects and safety are paramount in the development of cell-based immunotherapies [43]. Off-target effects occur when the engineered immune cells inadvertently target non-HIV-infected cells, leading to unintended tissue damage [43]. CAR T cells carry a risk of recognizing and attacking cells that express similar antigens to those of HIV. To mitigate this risk, researchers are designing CARs with higher specificity and employing safety switches that can be activated to eliminate the CAR T cells if severe adverse effects occur [43].

Cytokine release syndrome (CRS) is another potential complication associated with cell-based immunotherapies [44]. CRS is characterized by a massive release of inflammatory cytokines, which can lead to severe systemic inflammation and organ dysfunction [44]. The development of strategies to monitor and manage CRS is critical for the safe application of these therapies. Approaches such as the use of monoclonal antibodies to neutralize specific cytokines and the administration of corticosteroids to control inflammation are being explored to mitigate the risks associated with CRS [44].

Long-term safety is a crucial consideration, particularly given the potential for genetically modified cells to persist in the body for extended periods [45]. Ensuring the long-term safety of these therapies requires comprehensive monitoring for potential late-onset toxicities and the establishment of robust regulatory frameworks [45]. Preclinical studies and long-term follow-up in clinical trials are essential to assess the durability and safety of cell-based immunotherapies [45].

Recent scientific discoveries continue to drive the field forward. For example, the use of gene editing technologies, such as CRISPR-Cas9, to enhance the antiviral properties of immune cells is being actively explored [46]. Researchers are investigating ways to knock out genes that HIV exploits to evade the immune system or to insert genes that confer enhanced antiviral functions [46]. Combining gene editing with cell-based therapies holds the potential to create more effective and durable treatments [46].

Moreover, advances in synthetic biology are enabling the design of next-generation CARs and other engineered receptors with improved specificity and functionality [47]. These innovations include the development of dual-targeting CARs that recognize multiple HIV antigens, thereby reducing the risk of viral escape [47]. Synthetic biology approaches are also being used to create immune cells with programmable behaviors, such as the ability to secrete therapeutic agents in response to HIV-1 infection [47].

In conclusion, cell-based immunotherapies represent a promising avenue for HIV-1 treatment, with the potential to enhance antiviral immunity and target HIV-infected cells more effectively. While significant challenges remain, ongoing research and technological advancements are addressing these hurdles, bringing us closer to realizing the full potential of these therapies. Continued exploration of innovative strategies, combined with rigorous clinical testing, will be essential to achieving safe and effective cell-based immunotherapies for HIV-1.

## Challenges and Limitations

Despite the promising potential of stem cell therapies for HIV-1, several challenges and limitations need to be addressed to advance their clinical translation. One major hurdle is the high cost and complexity of these procedures. Stem cell therapies often involve sophisticated techniques and specialized infrastructure, making them financially inaccessible to many patients, particularly those in low-income countries or resource-limited settings [48]. This economic barrier is further exacerbated for pediatric patients, who may require additional considerations in treatment protocols and long-term follow-up care [48]. Ensuring global affordability and accessibility of these therapies is a significant challenge that requires innovative solutions and international cooperation (Table 2).

Another critical issue is the risk of adverse events associated with stem cell transplantation, such as graft-versus-host disease (GVHD) and genotoxicity [49]. GVHD occurs when the transplanted donor cells attack the recipient’s body, leading to severe complications and increased mortality [49]. Strategies to mitigate GVHD, such as using haploidentical donors or implementing more precise immune-matching techniques, are areas of active research [49]. Additionally, the potential for genotoxic effects, particularly with gene-edited cells, raises concerns about long-term safety [49]. Rigorous preclinical testing and long-term monitoring in clinical trials are essential to ensure that these therapies do not introduce new health risks [49].

Ethical considerations related to donor selection and informed consent are also paramount. The selection of suitable donors, particularly those with desirable genetic traits like the CCR5 delta-32 mutation, involves complex ethical and logistical challenges [50]. Informed consent processes must be robust and transparent, ensuring that donors and recipients fully understand the potential risks and benefits [50]. Addressing these ethical issues is critical to maintaining public trust and the ethical integrity of stem cell research and therapy (Table 2).

Furthermore, the heterogeneity of HIV-1 infection and the presence of viral reservoirs in sanctuary sites such as the gut, lymph nodes, and central nervous system (CNS) pose significant obstacles to achieving a functional cure [51]. HIV-1 can persist in these reservoirs despite effective cART, making it challenging to completely eradicate the virus [51]. Strategies to target and reduce latent viral reservoirs are a major focus of current HIV-1 research. These include latency-reversing agents that aim to “shock and kill” hidden viruses and enhance immune recognition and clearance of infected CD4^+^ T cells [51]. Achieving sustained viral suppression in the absence of cART requires a multifaceted approach, combining stem cell therapy with other therapeutic modalities to address the complex biology of HIV-1 [51].

## Future Directions

Future research directions in the field of stem cell therapy for HIV-1 should prioritize optimizing treatment protocols to enhance efficacy, tolerability and safety of the potent antiretroviral therapies in combination with other options. This includes refining gene editing techniques to increase specificity and reduce off-target effects, as well as improving the engraftment and persistence of transplanted cells. The development of more precise and efficient gene editing tools, such as CRISPR-Cas9, holds promise for creating HIV-resistant cells with minimal risk of adverse effects. Additionally, exploring combinatorial approaches with immunomodulatory agents or therapeutic vaccines could enhance the antiviral immune response and provide a synergistic effect in controlling HIV1 infection.

Large-scale clinical trials are essential to evaluate the long-term outcomes and durability of responses to stem cell-based therapies. These trials should include diverse populations to ensure that the findings are generalizable and applicable to different demographic groups, including pediatric and low-income populations. Collaborative efforts between researchers, clinicians, industry partners, and regulatory agencies are crucial to accelerating the development and implementation of these therapies. International partnerships and funding initiatives can help overcome economic and logistical barriers, facilitating the global dissemination of stem cell therapies for HIV-1. Moreover, future research should focus on innovative strategies to enhance the immune recognition and clearance of HIV-infected cells. This includes the development of novel CAR T-cells with enhanced specificity for HIV-1 antigens and the use of immune checkpoint inhibitors to boost T-cell function. Advances in synthetic biology and biomaterials could also contribute to creating more effective and durable cell-based therapies. For instance, the design of synthetic niches that support the survival and proliferation of transplanted cells *in vivo* could improve treatment outcomes.

## Conclusion

In conclusion, stem cell therapy holds immense promise as a potential curative strategy for HIV-1 by targeting viral reservoirs and restoring immune function. Significant progress has been made in preclinical and clinical studies, demonstrating the feasibility and potential efficacy of these approaches. However, challenges related to safety, efficacy, scalability, and cost-effectiveness remain significant obstacles that must be addressed. Ensuring global access to these therapies requires innovative solutions to reduce costs and simplify procedures, making them more accessible to patients in the low- middle income country (LMIC) settings and resource-limited environments.

Continued research efforts are needed to overcome these challenges and execution of the full potential of stem cell therapies for achieving HIV-1 remission or cure. This includes optimizing gene editing techniques, enhancing the immune response, and conducting large-scale clinical trials to evaluate long-term outcomes. Collaborative efforts between various stakeholders, including researchers, clinicians, pharma industry partners, and regulatory government agencies, are essential to advancing the HIV-1 cure field and translating promising findings into clinical practice.

Ultimately, the goal of stem cell therapy for HIV-1 cure is to achieve sustained viral suppression, eradication of persistent HIV reservoirs and immune reconstitution without the need for lifelong cART. While significant hurdles remain, the ongoing advancements in stem cell research and cART therapy provide a hopeful outlook for the future in the HIV field. That includes choosing a right treatment of cART with combination of the other options that are needed for the evaluation of the resident HIV-1 strains within PLWH so to minimize viral reservoirs, viral escape risks and to consider the implementation of a personalized treatment options. By addressing the current limitations and building on recent scientific discoveries related to the identification of the most potent, effective and safe bnAb cocktails that are a requirement on the future expressions of immunotherapy regimens, the potential for stem cell therapies to transform the treatment and management of HIV-1 is becoming increasingly tangible. Continued dedication to novel research, innovation, and collaboration will be key to unlocking the full potential of stem cell therapies and moving closer to an ultimate solution for HIV-1.

## Figures and Tables

**Table 1.** Summary of key points related to hematopoietic stem cell transplantation (HSCT) for HIV1. The table outlines crucial aspects, including the role of the CCR5 delta-32 mutation in achieving HIV-1 remission, the mechanisms underlying remission, challenges and risks associated with HSCT, donor availability issues, advancements in gene editing, future research directions, innovative therapeutic strategies, and the importance of patient education and support.

Section	Key Points
**CCR5 Delta-32 Mutation**	- CCR5 delta-32 mutation results in a truncated CCR5 protein, making immune cells less susceptible to HIV-1. - Cases like the “Berlin Patient” and “London Patient” show the potential for long-term HIV-1 remission through HSCT from CCR5 delta-32 homozygous donors.
**Mechanisms of Remission**	- Complete replacement of the host immune system with donor-derived cells resistant to HIV-1. - Graft-versus-host disease (GVHD) and conditioning regimens contribute to the reduction of the HIV-1 reservoir.
**Challenges and Risks**	- Transplant-related morbidity and mortality, including infections, organ toxicity, and GVHD. - Lifelong immunosuppressive therapy is often required, increasing susceptibility to complications.
**Donor Availability**	- Limited availability of suitable CCR5 delta-32 mutation donors, especially for ethnically diverse populations. - Need for expanding donor registries and exploring gene-editing technologies.
**Gene Editing and Future Directions**	- Advancements in CRISPR-Cas9 technology to modify autologous HSCs for HIV-1 resistance. - Research on non-myeloablative conditioning regimens and combination therapies. - Importance of large-scale clinical trials and personalized treatment approaches.
**Innovative Strategies And Biomarkers**	- Combining HSCT with latency-reversing agents and immune checkpoint inhibitors. - Development of biomarkers to monitor HSCT success and predict complications.
**Patient Education and Support**	- Essential for comprehensive patient counseling and continuous psychological support.

**Table 2.** Summary of challenges and limitations in advancing the clinical translation of stem cell therapies for HIV-1. The table highlights key points related to the high cost and complexity of procedures, the risk of adverse events such as graft-versus-host disease (GVHD) and genotoxicity, ethical considerations in donor selection and informed consent, and the heterogeneity of HIV-1 infection along with the presence of viral reservoirs.

Section	Key Points
**Cost and Complexity**	- High cost and complexity of stem cell therapies make them financially inaccessible to many patients, particularly in low-income countries or resource-limited settings. - Additional considerations are required for pediatric patients in treatment protocols and long-term follow-up care. - Ensuring global affordability and accessibility requires innovative solutions and international cooperation.
**Adverse Events**	- Risk of graft-versus-host disease (GVHD), where transplanted donor cells attack the recipient’s body, leading to severe complications and increased mortality. - Strategies to mitigate GVHD include using haploidentical donors and implementing more precise immune-matching techniques. - Potential for genotoxic effects, particularly with gene-edited cells, raises concerns about long-term safety. - Rigorous preclinical testing and long-term monitoring in clinical trials are essential to ensure safety.
**Ethical Considerations**	- Selection of suitable donors, especially those with desirable genetic traits like the CCR5 delta-32 mutation, involves complex ethical and logistical challenges. - Robust and transparent informed consent processes are necessary to ensure donors and recipients fully understand the risks and benefits. - Addressing ethical issues is critical to maintaining public trust and ethical integrity in stem cell research and therapy.
**Heterogeneity of HIV-1 and Viral Reservoirs**	- HIV-1 infection and the presence of viral reservoirs in sanctuary sites such as the gut and CNS pose significant obstacles to achieving a functional cure. - HIV-1 can persist in these reservoirs despite effective antiretroviral therapy (ART), making complete eradication challenging. - Strategies to target and reduce latent viral reservoirs include latency-reversing agents that aim to “shock and kill” hidden viruses and enhance immune recognition and clearance of infected CD4+ T cells. - Achieving sustained viral suppression in the absence of ART requires a multifaceted approach, combining stem cell therapy with other therapeutic modalities.
